# Prognostic value of low skeletal muscle mass in hepatocellular carcinoma patients treated with sorafenib or lenvatinib: A meta-analysis

**DOI:** 10.17179/excli2020-3111

**Published:** 2021-01-04

**Authors:** Jun Guan, Qin Yang, Chao Chen, Gang Wang, Haihong Zhu

**Affiliations:** 1 State Key Laboratory for Diagnosis and Treatment of Infectious Diseases, National Clinical Research Center for Infectious Diseases, Collaborative Innovation Center for Diagnosis and Treatment of Infectious Diseases, The First Affiliated Hospital, Zhejiang University School of Medicine

**Keywords:** low skeletal muscle mass, sorafenib, lenvatinib, hepatocellular carcinoma, prognosis

## Abstract

Growing evidence indicates that skeletal muscle depletion has a notable effect on the prognosis of hepatocellular carcinoma (HCC) patients, though study results are still controversial. Our meta-analysis aimed at evaluating the prognostic significance of low skeletal muscle mass (LSMM) in HCC patients treated with sorafenib or lenvatinib.We systematically reviewed for PubMed, Cochrane, and Embase databases from their inception to August 2020 and obtained all relevant articles describing an association between LSMM and HCC patients treated with sorafenib or lenvatinib. Demographic and characteristics of included studies, diagnostic criteria of skeletal muscle depletion, and main outcomes (overall survival, progression-free survival, time to treatment failure) were retrieved. Associations were expressed by calculating hazard ratios (HRs) and 95 % confidence intervals (CIs).The meta-analysis enrolled 11 studies comprising 1148 patients. Without significant heterogeneity between studies, LSMM was significantly associated with poor overall survival (crude HR=1.58, 95 % CI: 1.36-1.83; adjusted HR=1.83, 95 % CI: 1.46-2.29) and time to treatment failure (crude HR=1.85, 95 % CI: 1.34-2.54; adjusted HR=1.72, 95 % CI: 1.24-2.38). However, there was no significantly association between LSMM and progression-free survival (adjusted HR=1.44, 95 % CI: 0.95-2.20). Symmetry of distribution on the funnel plot did not show significant publication bias.This meta-analysis supported that LSMM is significantly associated with poor overall survival and time to treatment failure in HCC patients after sorafenib or lenvatinib administration. This negative effect was pronounced even after adjustment for confounders. Future studies should be carried out on larger samples and study regions based on standardized thresholds of LSMM.

## Introduction

Hepatocellular carcinoma (HCC), characterized by high incidence and high mortality, is the sixth most malignant tumor and ranks fourth in the list of causes of cancer-related death globally (Ferlay et al., 2019[12]). Especially in Africa and East Asia, HCC has caused severe economic and health care burdens. Due to inconspicuous symptoms of early HCC, a large majority of patients are not diagnosed with it until advanced stages. They tend to have restricted treatment options and poor outcomes.

Sorafenib, an oral kinase inhibitor, can simultaneously inhibit molecules and pathways relevant to tumor proliferation and angiogenesis (Wilhelm et al., 2004[42]). It was firstly recommended as the first-line treatment for advanced HCC patients that are refractory to locoregional therapy, resection, or transplantation. Compared to placebo, sorafenib is beneficial in prolonging time to progression and median overall survival (OS) (Cheng et al., 2009[6]; Keating, 2017[21]). Later, lenvatinib, another tyrosine kinase inhibitor (TKI), demonstrated a comparable efficacy to sorafenib and was even superior in increasing progression-free survival (PFS) (Kudo et al., 2018[22]), thus was approved for the second first-line drug by the National Medical Products Administration (NMPA) in September 2018. Regardless of their remarkable efficacy, adverse effects cannot be neglected, such as renal toxicity, fatigue, diarrhea, hand-foot skin reaction, weight loss and hypertension, and may result in dose reduction or discontinuation under severe conditions. These adverse events accelerate disease progression and shorten survival by muscle depletion (Antoun et al., 2010[4]). Thus, we should pay more attention to the changes in body composition during sorafenib administration.

Skeletal muscle depletion, termed as sarcopenia, is defined by progressive and generalized loss of muscle mass and muscle function (Cruz-Jentoft and Sayer, 2019[8]), which is related to aging, nutritional disorders, or some underlying diseases. Loss of skeletal muscle mass contributes to cancer-associated cachexia and further seriously threatens the quality of life and survival. Reversing sarcopenia markedly ameliorates the quality of life in breast cancer patients (Adams et al., 2016[1]). An increasing number of studies focus on the relationship between skeletal muscle depletion and poor outcomes in malignancies. Therefore, our meta-analysis intended to evaluate the prognostic importance of low skeletal muscle mass (LSMM) in unresectable HCC patients treated with the first-line TKIs.

## Materials and Methods

### Search strategies

Electronic databases involving PubMed, Embase, and Cochrane Library were searched and browsed to obtain all eligible articles without any restrictions on publication language and year. The following terms were employed to complete search function: (“sorafenib” OR “Nexavar” OR “lenvatinib” OR “lenvima” OR “tyrosine kinase inhibitors” OR “TKIs”) AND (“sarcopenia” OR “skeletal muscle” OR “muscle depletion”) AND (“hepatocellular carcinoma” OR “liver cancer” OR “liver cell carcinoma” OR “hepatoma” OR “HCC”). We also examined the reference lists of satisfied publications to search for more relevant citations.

### Inclusion and exclusion criteria

Eligible studies needed to meet the following criteria: (1) retrospective or prospective studies (2) treated with sorafenib or lenvatinib rather than other kinase inhibitors (3) the outcome was OS, PFS or time to treatment failure (TTF) and (4) provided hazard ratios (HRs) and 95 % confidence intervals (CIs). Case reports, review articles, duplicate literature, and studies involving other kinase inhibitors or without any outcome of interest were excluded. When it came to studies with overlapped patient data, we chose the one involving the largest sample size and the longest duration.

### Data extraction and quality assessment

Two authors (JG and QY) independently collected data using specially-designed electronic forms. The following details were extracted: first author's name, publication year, title, country, study design, enrolled numbers (male vs female), HCC stage, age, prevalence of LSMM, details about measured muscle, cut-off value for LSMM, outcome variables (OS, PFS and TTF), and adjustment factors. OS was defined as the interval from the initial date of TKIs administration to the date of death or last follow-up. PFS was defined as the interval from the initial date of TKIs administration to the date of death, disease progression or last follow-up. TTF was defined as the interval from treatment initiation to the end or last follow-up.

The quality evaluation of the involved studies was performed by using the Newcastle-Ottawa Scale (NOS). Studies were scored based on three major criteria: the selection of the study groups (four items); the comparability of the groups (one item); and the ascertainment of either the outcome or exposure of interest for cohort or case-control studies respectively (three items). The maximum score of the NOS was 9 points. Studies with scores of more than 6 points were considered to be of high quality; less than 4 points of low quality; while those with scores of 4 to 6 were of medium quality.

### Statistical analysis

The outcomes for the association between LSMM and OS, PFS or TTF were expressed as crude and adjusted HRs with 95 % CIs. HRs and 95 % CIs were obtained directly from univariate and multivariate COX regression analyses and needed to be further converted into natural logarithm (lnHR) and standard error (SE). We assessed heterogeneity by using Cochran's Q statistic, with p < 0.1 and I^2 ^> 50 % being suggestive of meaningful heterogeneity (Higgins et al., 2003[14]). When heterogeneity was observed, the random-effects model was selected; otherwise the fixed-effects model was utilized. Potential publication bias was evaluated by using funnel plots. All calculations were performed using Review Manager 5.3, and p < 0.05 was considered statistically significant.

## Results

### Search results

**Figure 1:**
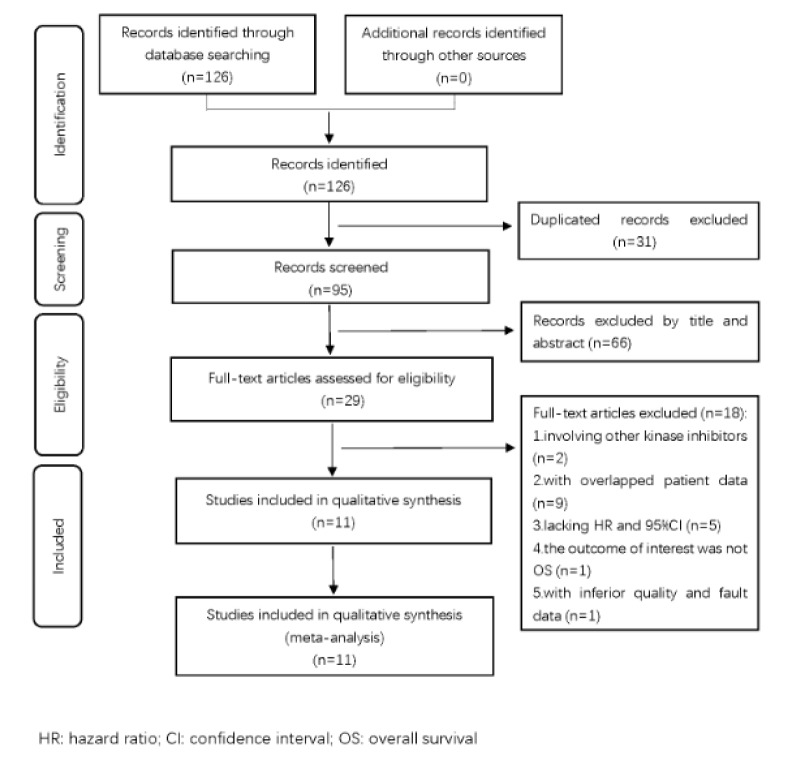
The flow diagram of this study selection process

Of the 126 studies identified through database searching, 31 duplicated studies were excluded and 95 studies were screened. After being excluded by titles and abstracts, 29 full-text articles were assessed for eligibility. 18 records didn't meet the inclusion criteria and were discarded: 2 involving other kinase inhibitors (Nault et al., 2013[28], 2015[29]), 9 with overlapped patient data (Antonelli et al., 2018[2]; Gigante et al., 2015[13]; Hoshino et al., 2015[16]; Imai et al., 2015[17], 2019[19]; Labeur et al., 2018[23][24]; Okada et al., 2019[33]; Saeki et al., 2019[34]), 5 lacking HR and 95 % CI (Mir et al., 2012[26]; Okada et al., 2020[32]; Saeki et al., 2018[35]; Uchikawa et al., 2020[39]; Ueki et al., 2016[40]), 1 without any interesting outcome (Cheng et al., 2019[7]) and 1 with low quality and faulty data (Nishikawa et al., 2017[30]). Thus, 11 retrospective studies (Antonelli et al., 2018[3]; Endo et al., 2020[10]; Hiraoka et al., 2017[15]; Imai et al., 2020[18]; Labeur et al., 2019[25]; Naganuma et al., 2017[27]; Sawada et al., 2019[36]; Takada et al., 2018[38]; Uojima et al., 2020[41]; Wu et al., 2021[43]; Yamashima et al., 2017[45]) were included in this meta-analysis, comprising 1148 patients. The flow diagram of this study selection process is shown in Figure 1(Fig. 1). 

### Characteristics of included studies

The demographic and characteristics of the 11 eligible studies are shown in Table 1(Tab. 1) (References in Table 1: Antonelli et al., 2018[3]; Endo et al., 2020[10]; Hiraoka et al., 2017[15]; Imai et al., 2020[18]; Labeur et al., 2019[25]; Naganuma et al., 2017[27]; Sawada et al., 2019[36]; Takada et al, 2018[38]; Uojima et al., 2020[41]; Wu et al., 2020[43]; Yamashima et al., 2017[45]). Out of these retrospective studies, 9 were performed in Asia (8 in Japan and 1 in China) and the remaining 2 in Europe (Netherlands and Italy). The vast majority of the HCC patients were in an advanced stage. The median ages of participants ranged from 64 to 72 years. Male patients accounted for the vast majority of all the participants. All diagnoses were made through computed tomography scans, but the measured indicators and cut-off values for LSMM varied. In 8 studies, the total skeletal muscle (TSM) mass was quantified at the third lumbar level (L3). One study measured the psoas muscle (PM) mass at L3 and 1 study measured transverse psoas muscle thickness at the level of the umbilicus. Moreover, 1 study defined LSMM based on TSM, PM, and rectus abdominis (RA) indices respectively. After excluding this study without reported number (Wu et al., 2021[43]), the frequency of LSMM patients reached 41 %. The results of the quality evaluation are demonstrated in Table 2(Tab. 2) (References in Table 2: Antonelli et al., 2018[3]; Endo et al., 2020[10]; Hiraoka et al., 2017[15]; Imai et al., 2020[18]; Labeur et al., 2019[25]; Naganuma et al., 2017[27]; Sawada et al., 2019[36]; Takada et al, 2018[38]; Uojima et al., 2020[41]; Wu et al., 2020[43]; Yamashima et al., 2017[45]), and all studies were regarded as being of high quality.

### Overall survival

**Figure 2:**
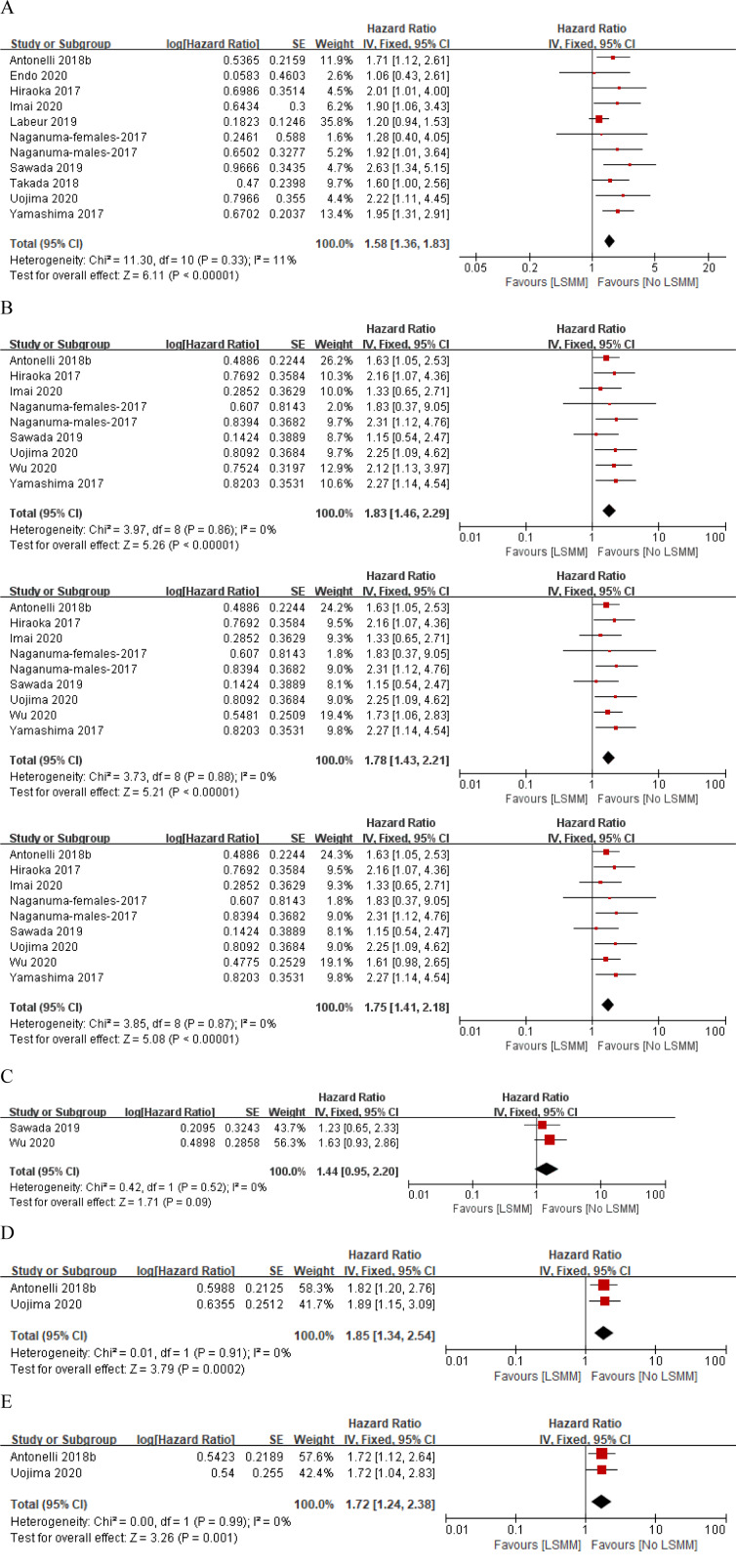
Forest plot evaluating the association between the low skeletal muscle mass and hepatocellular carcinoma patients treated with sorafenib or lenvatinib: crude (A) and adjusted (B) HRs between low skeletal muscle mass and overall survival, adjusted HR between low skeletal muscle mass and progression-free survival (C), crude (D) and adjusted (E) HRs between low skeletal muscle mass and time to treatment failure.

**Figure 3:**
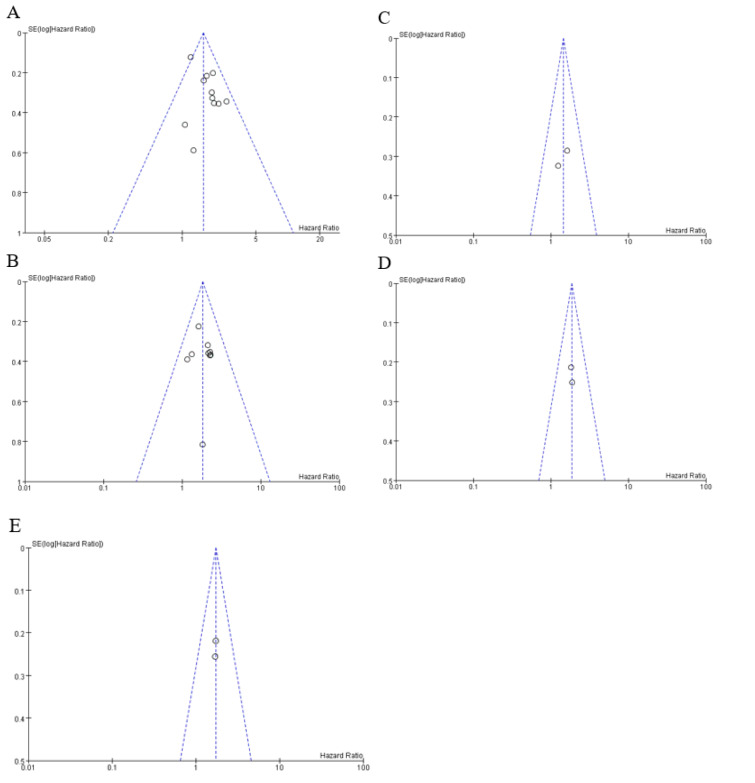
Publication bias analysis by using funnel plot for crude (A) and adjusted (B) HRs of overall survival, adjusted (C) HR of progression-free survival, crude (D) and adjusted (E) HRs of time to treatment failure.

The main results of the crude and adjusted pooled analysis are reported in Figure 2A and 2B(Fig. 2) (References in Figure 2: Antonelli et al., 2018[3]; Endo et al., 2020[10]; Hiraoka et al., 2017[15]; Imai et al., 2020[18]; Labeur et al., 2019[25]; Naganuma et al., 2017[27]; Sawada et al., 2019[36]; Takada et al, 2018[38]; Uojima et al., 2020[41]; Wu et al., 2020[43]; Yamashima et al., 2017[45]) respectively. Ten studies involving 1028 patients provided the crude HRs and 95 % CIs of the association between the LSMM and HCC patients treated with sorafenib or lenvatinib (Antonelli et al., 2018[3]; Endo et al., 2020[10]; Hiraoka et al., 2017[15]; Imai et al., 2020[18]; Labeur et al., 2019[25]; Naganuma et al., 2017[27]; Sawada et al., 2019[36]; Takada et al., 2018[38]; Uojima et al., 2020[41]; Yamashima et al., 2017[45]). A fixed-effects model was utilized with no significant heterogeneity (p value=0.33; I^2 ^= 11 %). The crude pooled HR was 1.58 (95 % CI 1.36, 1.83; p < 0.00001) and supported the association between LSMM and poor prognosis (Figure 2A(Fig. 2)). Eight studies involving 661 patients provided the adjusted HRs and 95 % CIs (Antonelli et al., 2018[3]; Hiraoka et al., 2017[15]; Imai et al., 2020[18]; Naganuma et al., 2017[27]; Sawada et al., 2019[36]; Uojima et al., 2020[41]; Wu et al., 2021[43]; Yamashima et al., 2017[45]). One of these studies defined LSMM based on TSM, PM, and RA indices and provided three different corresponding HRs (Wu et al., 2021[43]), so three adjusted pooled results were obtained. There was no heterogeneity (p value = 0.9; I^2 ^= 0 %) regardless of the three different HRs, so we conducted a forest plot by applying a fixed-effects model. The adjusted pooled HRs were 1.83 (95 % 1.46, 2.29; p < 0.00001), 1.78 (95 % 1.43, 2.21; p < 0.00001), and 1.75 (95 % 1.41, 2.18; p<0.00001) respectively (Figure 2B(Fig. 2)). Symmetry of distribution on the funnel plot indicated that there was no publication bias (Figure 3A and Figure 3B(Fig. 3)).

**Figure 4:**
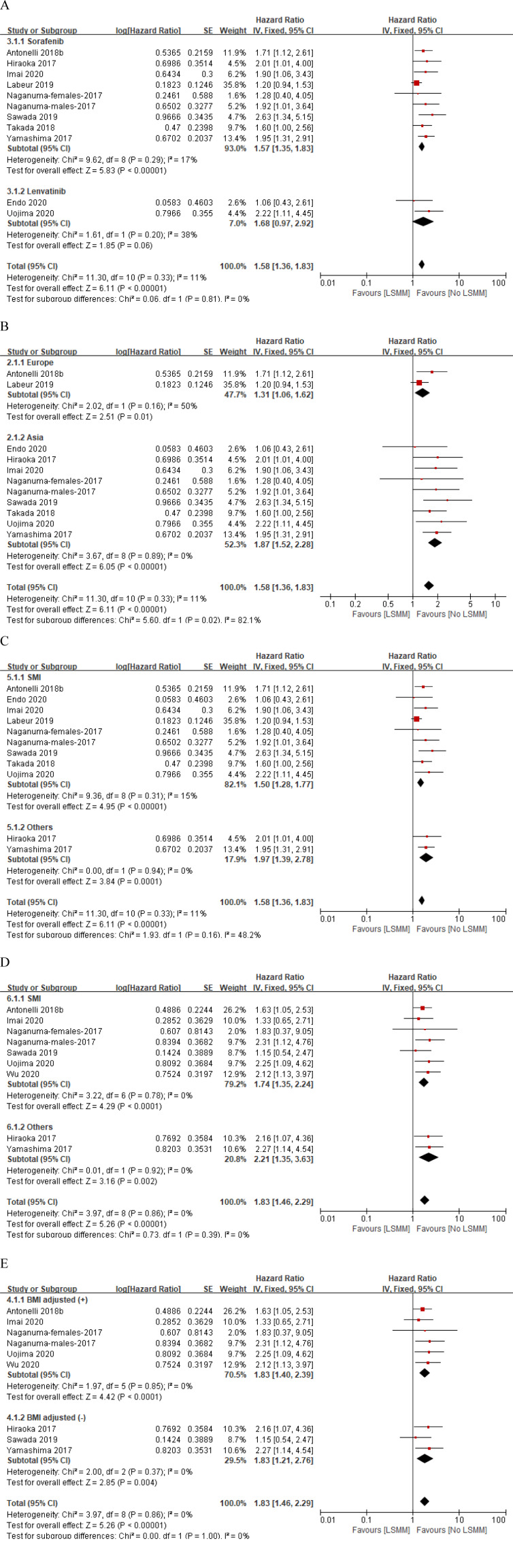
Forest plot of the subgroup analyses for the crude association between low skeletal muscle mass and overall survival stratified by types of TKIs (sorafenib or lenvatinib) (A), by study region (Europe and Asia) (B) and by muscle measured (SMI or others) (C); the adjusted association between low skeletal muscle mass and overall survival stratified by muscle measured (SMI or others) (D), by whether body mass index or underweight or body weight was involved in multivariate analysis (BMI adjusted [+] or BMI adjusted [-]) (E). BMI, body mass index; SMI, skeletal muscle index (cm^2^/m^2^)

To further investigate the association between LSMM and prognosis in HCC patients with the first-line TKIs administration, we conducted subgroup analyses stratified by types of TKIs (sorafenib or lenvatinib), study region (Europe and Asia), muscle measured (skeletal muscle index [SMI] or others) and whether body mass index (BMI) or underweight or body weight was involved in multivariate analysis (BMI adjusted [+] or BMI adjusted [-]). When stratifying by types of TKIs, we found significantly negative impact of LSMM on OS in patients treated with sorafenib (p<0.00001), but there was no significant association in patients treated with lenvatinib (p=0.06), probably because of the small number of included studies (n=2) (Figure 4A(Fig. 4); References in Figure 4: Antonelli et al., 2018[3]; Endo et al., 2020[10]; Hiraoka et al., 2017[15]; Imai et al., 2020[18]; Labeur et al., 2019[25]; Naganuma et al., 2017[27]; Sawada et al., 2019[36]; Takada et al, 2018[38]; Uojima et al., 2020[41]; Wu et al., 2020[43]; Yamashima et al., 2017[45]). Then, we did not find other subgroup analyses to be a significant effect modifier for the association between LSMM and OS (Figure 4(Fig. 4)). At least, all above results firmly supported that LSMM could be a poor prognostic factor for OS in HCC patients after sorafenib administration.

### Progression-free survival

Only two studies involving 202 patients reported the adjusted HRs and 95 % CIs of the association between the LSMM and HCC patients treated with sorafenib or lenvatinib (Sawada et al., 2019[36]; Wu et al., 2021[43]). One defined LSMM based on TSM, PM, and RA indices and provided three different corresponding HRs, the other is based on TSM, so we uniformly use the HR corresponding to the TSM index. A fixed-effects' model was utilized with no significant heterogeneity (p value = 0.52; I ^2^= 0 %). The crude pooled HR was 1.44 (95 % CI 0.95, 2.20; p = 0.09) and supported that there is no significant association between LSMM and PFS (Figure 2C(Fig. 2)). The symmetrical distribution on the funnel plot indicated that there was no publication bias. The stratified analysis was not conducted owing to the limited number of studies involving PFS (Figure 3C(Fig. 3)).

### Time to treatment failure

Likewise, only two studies involving 196 patients reported the crude and adjusted HRs and 95 % CIs of the association between the LSMM and HCC patients treated with sorafenib or lenvatinib (Antonelli et al., 2018[3]; Uojima et al., 2020[41]). A fixed-effects' model was utilized with no significant heterogeneity (p value > 0.5; I^2 ^= 0 %). LSMM was significantly associated with TTF with a crude pooled HR of 1.85 (95 % CI 1.34-2.54; p = 0.0002) and an adjusted pooled HR of 1.72 (95 % CI 1.24-2.38; p = 0.001) (Figure 2D and Figure 2E(Fig. 2)). Symmetry of distribution on the funnel plot supported no evidence of publication bias. The stratified analysis was not conducted because of the limited number of studies involving TTF (Figure 3D and Figure 3E(Fig. 3)).

## Discussion

Our meta-analysis paid attention to the impact of LSMM on the prognosis of HCC patients treated with the first-line TKIs for the first time. Based on 11 studies and 1148 patients, we found that LSMM has a negative effect on OS and TTF, but has no significant impact on PFS. Even after adjusting for relevant confounders, this correlation about OS and TTF remained pronounced. Except for the subgroups stratified by types of TKIs, the pooled results for the remaining subgroup analyses were not observably influenced. Our results supported that LSMM may be a promising poor prognosis for outcomes in HCC patients treated with the first-line TKIs.

Reported studies demonstrated that skeletal muscle mass is associated with the prognosis of multiple malignancies and postoperative complications of HCC. A meta-analysis of 38 studies demonstrated that LSMM was correlated with poor OS in multiple solid tumors (involving HCC) (Shachar et al., 2016[37]). Chang et al. further conducted a meta-analysis including 13 HCC studies, and concluded that sarcopenia was associated with increased all-cause mortality and tumor recurrence in HCC patients (Chang et al., 2018[5]). However, due to the limited number of studies, the above studies did not perform stratified analyses based on tumor stage. Considering that advanced HCC patients often present with skeletal muscle depletion and existing studies have a dispute over the relationship between LSMM and prognosis after sorafenib introduction, so it is of great clinical significance to validate the relationship based on the latest research.

LSMM was prevalent in HCC patients treated with sorafenib or lenvatinib in our articles, with reported prevalence rates ranging from 20 % to 59 %. The potential mechanisms are as follows (Antoun et al., 2010[4]; Nishikawa et al., 2016[31]): (1) Insufficient glycogen storage. To compensate for glycogen depletion, skeletal muscles degrade to provide glucose and amino acids (such as branched-chain amino acids, BCAA) and result in a decrease in blood BCAA. Thus, the function of BCAA as the strongest material for protein synthesis to maintain and increase muscle mass is hindered; (2) Impaired synthesis of insulin growth factor 1 (IGF-1). IGF-1 aims to maintain the dynamic balance between protein anabolism and catabolism; (3) Increased level of blood myostatin. Myostatin is a member of the transforming growth factor β (TGF-β) family and can strongly inhibit skeletal muscle growth; (4) Up-regulated inflammatory cytokines and reactive oxygen species. Tumor necrosis factor-α (TNF-α) and interleukin-6 (IL-6) can accelerate protein catabolism. Reactive oxygen species can inhibit protein anabolism; (5) Sorafenib can suppress muscle protein synthesis directly by inhibiting mTOR phosphorylation that triggers muscle protein synthesis under activated conditions. Compared to controls, LSMM is significantly associated with an increased risk of mortality. High levels of tumor necrosis factor-α (TNF-α) and interleukin-6 (IL-6) play an indispensable role. TNF-α, act as an important regulator of the tumor microenvironment, can promote tumor migration and invasion by the TNF-α-NF-κB-Snail pathway (Wu and Zhou, 2010[44]). Over-expression of IL-6 activates hepatocarcinogenesis and deteriorates liver function through p-STAT3 (Kao et al., 2015[20]). Sorafenib and lenvatinib, as the first-line drugs for advanced liver cancer, are recognized to inhibit tumor proliferation and angiogenesis, thus prolonging survival. On the other hand, sorafenib inhibits skeletal muscle protein synthesis and may lead to LSMM. The reduction of skeletal muscle mass is considered one of the criteria for diagnosing both sarcopenia and cancer cachexia (Fearon et al., 2011[11]; Nishikawa et al., 2016[31]). Sarcopenia and cancer cachexia may lead to LSMM and poor prognosis. Actually, patients with LSMM tend to have a shorter administration duration of sorafenib due to serious adverse reactions. Therefore, it is recommended to carry out prospective studies aiming to investigate whether sorafenib and lenvatinib can benefit the survival of LSMM patients.

Early evaluation and adequate intervention of high-risk factors can improve the prognosis. Efficient treatment of LSMM includes exercise, nutritional support and pharmacological agents (Dutt et al., 2015[9]; Nishikawa et al., 2016[31]). After exercise, IGF-1 synthesized by hepatocytes and myocytes is up-regulated. Nutritional support, such as BCAA, contributes to protein synthesis and increased skeletal muscle mass. The restored skeletal muscle mass can prolong the duration of the first-line TKIs administration, improve survival time and the quality of life of patients with cachexia at end-stage.

Undeniably, there are several limitations to our meta-analysis. Firstly, the articles involved were retrospective, with limited numbers of participants and just a few regions. Retrospective assessment of the outcome could be associated with selection bias and reporting bias. As such, a prospective study including a larger sample size in multiple centers should be conducted. Secondly, there were limited articles to enable stratified analysis based on the study region and types of TKIs. Due to the difference in the cut-off value and the basic characteristics of the population, a combined analysis is not the best. Thirdly, measured muscles and cut-off values that defined LSMM of all included articles vary considerably between Asia and Europe. Cut-off values for LSMM may be gender-specific and weight-specific. Differences in cut-off values of LSMM have an impact on the results. It is necessary to reach an international consensus on the diagnostic criteria of LSMM as soon as possible. Lastly, the initial doses of sorafenib were different between different cohorts, and this may have caused some bias.

## Conclusion

Based on this meta-analysis, we concluded that LSMM is associated with poor OS and TTF in HCC patients treated with sorafenib or lenvatinib. This negative effect was enhanced even after adjustment for confounders. Shortly, we should enlarge the sample and study more regions based on the standardized threshold of LSMM when performing more prospective studies. It is equally important to validate whether LSMM patients can benefit from sorafenib or lenvatinib treatment. After all, the ultimate goal of all the therapy is to maximize the benefits of patients with end-stage malignancies.

## Funding

This study was supported by grants awarded by the National Science and Technology Major Project of China (NO 2018ZX10302206), Science and Technology Major Projects of Zhejiang Province (NO 2018C04016).

## Conflict of interest

All authors declare that they have no conflict of interest. 

## Figures and Tables

**Table 1:**
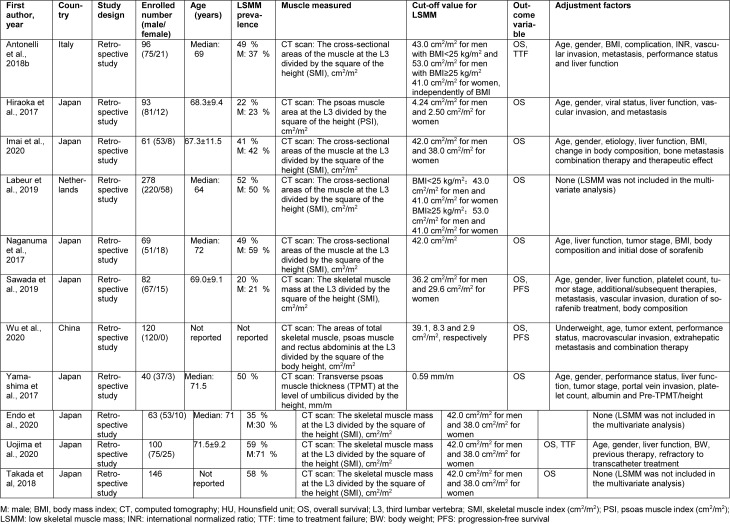
Demographic and characteristics of included studies

**Table 2:**
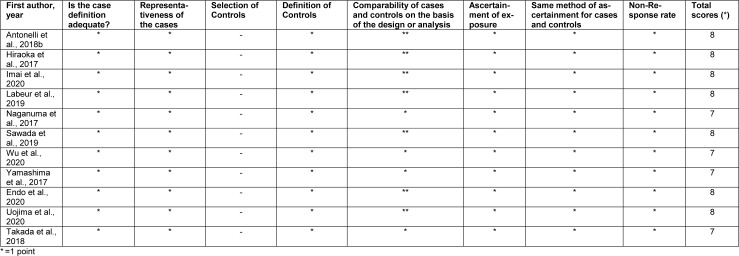
Quality assessment by using The Newcastle-Ottawa Scale
